# Thermic effect of soy versus whey protein – a pilot trial

**DOI:** 10.1186/1550-2783-9-S1-P26

**Published:** 2012-11-19

**Authors:** Bianca Rubin, Joseph Hashim, Sandra Sharp, Jose Antonio

**Affiliations:** 1 Nova Southeastern University, Davie, FL, 33314, USA

## Background

Protein has a thermic effect that exceeds both fat or carbohydrate. However, it is unclear if there is a difference in the thermic effect of feeding (TEF) between different protein sources. Thus, the purpose of this study was to determine the thermic effect of an isocaloric feeding of whey versus soy protein.

## Methods

In this single blind cross-over study, young male and female subjects (n=5, three males, two females; age range 18-21) consumed 40 grams of either whey (Zero Carb SRO by VPX) or soy protein (Iso-Rich Soy by Jarrow Formulas). Subjects reported to the lab on separate days (with at least 2 days between testing sessions) and underwent 3 hours of resting metabolic rate (RMR) testing. The thermic effect of feeding (TEF) was assessed via oxygen uptake measures at baseline and 1, 2, and 3 hours post-consumption of protein. Data was collected via the ParvoMedics metabolic cart.

## Results

A paired t-test for AUC reveled a 14.54% greater TEF for the whey protein than soy (p <0.05). The range amongst the subjects was 4.05%-23.36% greater increase in TEF. The average peak in oxygen uptake was 29.94% for whey protein and 23.98% for soy protein, respectively.

## Conclusion

Based on this small sample size, there is evidence to suggest that whey protein may have a greater TEF than soy.

